# Supplementary material for: Multi‐dataset identification of innovative feature genes and molecular mechanisms in keratoconus
Source: J Cell Mol Med. 2024 Sep 19;28(18):e70079. doi: 10.1111/jcmm.70079 (PMC11412914; doi:10.1111/jcmm.70079)
Supplement: Supplementary file 2 — Material S2. [file JCMM-28-e70079-s002.zip › 7.GSEA analysis/3.DBNDD1/KEGG_FRUCTOSE_AND_MANNOSE_METABOLISM.html]

Details for gene set KEGG\_FRUCTOSE\_AND\_MANNOSE\_METABOLISM[GSEA]

|  || Dataset | DBNDD1.group.cls#h\_versus\_l |
| Phenotype | group.cls#h\_versus\_l |
| Upregulated in class | l |
| GeneSet | KEGG\_FRUCTOSE\_AND\_MANNOSE\_METABOLISM |
| Enrichment Score (ES) | -0.4026961 |
| Normalized Enrichment Score (NES) | -1.1430773 |
| Nominal p-value | 0.33677685 |
| FDR q-value | 0.69121534 |
| FWER p-Value | 0.995 |
Table: GSEA Results Summary

  

Fig 1: Enrichment plot: KEGG\_FRUCTOSE\_AND\_MANNOSE\_METABOLISM      
 Profile of the Running ES Score & Positions of GeneSet Members on the Rank Ordered List

  

| SYMBOL | TITLE | RANK IN GENE LIST | RANK METRIC SCORE | RUNNING ES | CORE ENRICHMENT || 1 | ALDOC | na | 5665 | 0.159 | -0.1089 | No |
| 2 | SORD | na | 7617 | 0.112 | -0.1271 | No |
| 3 | PMM2 | na | 7933 | 0.105 | -0.1003 | No |
| 4 | MTMR7 | na | 8758 | 0.090 | -0.0934 | No |
| 5 | FBP1 | na | 9407 | 0.080 | -0.0849 | No |
| 6 | PHPT1 | na | 13551 | 0.034 | -0.1928 | No |
| 7 | FPGT | na | 14086 | 0.029 | -0.1982 | No |
| 8 | PFKP | na | 15214 | 0.021 | -0.2234 | No |
| 9 | MPI | na | 17171 | 0.008 | -0.2770 | No |
| 10 | PFKL | na | 17306 | 0.007 | -0.2783 | No |
| 11 | MTMR2 | na | 19539 | -0.009 | -0.3396 | No |
| 12 | MTMR6 | na | 19647 | -0.010 | -0.3393 | No |
| 13 | TPI1 | na | 19904 | -0.012 | -0.3425 | No |
| 14 | GMPPA | na | 20327 | -0.016 | -0.3493 | No |
| 15 | HK1 | na | 21104 | -0.023 | -0.3638 | No |
| 16 | ALDOA | na | 21556 | -0.027 | -0.3674 | No |
| 17 | PFKM | na | 22338 | -0.035 | -0.3778 | No |
| 18 | MTMR1 | na | 22444 | -0.037 | -0.3684 | No |
| 19 | PFKFB2 | na | 22506 | -0.037 | -0.3574 | No |
| 20 | PFKFB4 | na | 24078 | -0.057 | -0.3831 | Yes |
| 21 | GMDS | na | 24229 | -0.059 | -0.3672 | Yes |
| 22 | GMPPB | na | 24420 | -0.062 | -0.3515 | Yes |
| 23 | PMM1 | na | 25770 | -0.083 | -0.3620 | Yes |
| 24 | KHK | na | 26140 | -0.090 | -0.3418 | Yes |
| 25 | HK3 | na | 26158 | -0.091 | -0.3113 | Yes |
| 26 | PFKFB3 | na | 26896 | -0.105 | -0.2965 | Yes |
| 27 | AKR1B1 | na | 28153 | -0.134 | -0.2868 | Yes |
| 28 | ALDOB | na | 28498 | -0.143 | -0.2478 | Yes |
| 29 | HK2 | na | 29828 | -0.183 | -0.2237 | Yes |
| 30 | FBP2 | na | 31299 | -0.233 | -0.1866 | Yes |
| 31 | PFKFB1 | na | 33250 | -0.349 | -0.1237 | Yes |
| 32 | AKR1B10 | na | 34307 | -0.487 | 0.0121 | Yes |
Table: GSEA details [plain text format]

  

Fig 2: KEGG\_FRUCTOSE\_AND\_MANNOSE\_METABOLISM      
 Blue-Pink O' Gram in the Space of the Analyzed GeneSet

  

Fig 3: KEGG\_FRUCTOSE\_AND\_MANNOSE\_METABOLISM: Random ES distribution      
 Gene set null distribution of ES for **KEGG\_FRUCTOSE\_AND\_MANNOSE\_METABOLISM**
